# Mobile Measurement of PM_2.5_ Based on an Individual in Ulaanbaatar City

**DOI:** 10.3390/ijerph17082701

**Published:** 2020-04-15

**Authors:** Ariundelger Ariunsaikhan, Sonomdagva Chonokhuu, Yutaka Matsumi

**Affiliations:** 1 Department of Environment and Forest Engineering, School of Engineering and Applied Science, National University of Mongolia, Ulaanbaatar 210646, Mongolia; 16B1SEAS0378@stud.num.edu.mn; 2 Institute for Space-Earth Environmental Research, Nagoya University, Chikusa-ku, Nagoya 464-8601, Japan; matsumi@nagoya-u.jp

**Keywords:** PM_2.5_, mobile measurements, personal exposure, dose, micro environments

## Abstract

In the present study, we measured fine particulate matter (PM_2.5_) on the daily route of our study participant in order to determine her exposure and dose of PM_2.5_ in every microenvironment (ME). The measuring instrument, created by Nagoya University and Panasonic Corporation, Japan, was carried close to the breathing zone most of the time. Each data point was collected for 10–30 s or 2–6 cycles/min for 24 h from 1 October 2018 to 30 December 2018. Public transportation showed the highest level of PM_2.5_ compared with other MEs, including residence apartments, houses (ger district), the National University of Mongolia (NUM), food courts or restaurants, and other indoor locations. The personal daily average exposure to PM_2.5_ was 35 µg/m^3^ on 4 November 2018; on the other hand, this value was evaluated as the highest level of exposure compared to other measurement days. Interestingly, the study participant‘s exposure and dose of PM_2.5_ was lower than those stated in the World Health Organization (WHO) air quality guidelines, with 25 µg/m^3^ from 4:00 to 7:00.

## 1. Introduction

According to the global ranking of mortality risk factors, air pollution is the fifth highest risk factor and ranks higher than well-known hazardous components, such as alcohol use, occupational risk, and physical inactivity [1]. The World Health Organization (WHO) announced that nine out of 10 people breathe air containing a high level of pollutants [2,3]. The diameter of PM_2.5_, one of the major pollutants of air pollution, is less than 2.5 micrometres; however, it is capable of carrying various toxic materials. When humans breathe, PM_2.5_ enters the human body through air exchange and reaches the ends of the pulmonary alveoli, thereby damaging other parts of the body [4,5]. Primary sources of PM_2.5_ can be incomplete fuel combustion, biomass burning, vehicle exhaust, residential cooking, and bioaerosols [6]. The adverse effects of combustion-related air pollution are premature death, pulmonary diseases, including asthma, and an increased risk of developing cancer [7,8,9]. Alexander Millman (2008) from Columbia University suggests that PM_2.5_ causes micro-inflammation to a newborn’s brain [10].

The average daily temperature in Ulaanbaatar (UB), the capital city of Mongolia, is around −13 °C and sometimes reaches temperatures as low as −40 °C at night in the winter [11]. As of 2010, the population of UB was 1.24 million, but the number grew to 1.50 million by 2018 [12]. This population growth has led to major increases in the city’s air pollution emissions, as 53% of UB citizens live in the ger (the traditional Mongolian dwelling) areas, where coal and other flammable fuels are used for their heating systems [13]. The Mongolian National Agency for Meteorology and Environment Monitoring reports that, in 2017, in the wintertime, the mean concentration of particulate matter for the country as a whole was between 80–140 µg/m^3^ [14]. Additionally, diseases such as pneumoconiosis (approximately 130 cases per year) and adult cardiovascular diseases (approximately 1440 cases per year) that are caused by PM_2.5_ (70 μg/m^3^, the annual mean exposure) are the major morbidity causes for UB‘s population, with an increasing rate over the years [15]. 

Determining the individual exposure and dose of PM_2.5_ is also important for human health problems. There is a high correlation between indoor and outdoor locations and individual exposure [16,17,18,19]. Individual exposure is defined by the PM_2.5_ concentration of indoor or outdoor locations and personal activities, such as cooking, cleaning, and smoking, as well as the time spent by the person in the environment [20,21]. One of the methods used to evaluate the exposure is a measuring monitor or sensor worn by a person (the monitor or sensor has to be as close as possible to the person’s breathing zone [22,23,24]) in order to identify the interface between outdoor locations or various microenvironments (closed spaces, such as buildings, means of transportation, and other indoor locations [25]) and the body. The dose, depending on a human’s breathing speed, is the amount of the pollutant that actually crosses one of the body’s boundaries and reaches the target tissue [26]. 

In Mongolia, this kind of study has not been conducted before. However, researchers from other countries, for example, Steinle (2012), have conducted studies in this research field. Their results showed that a total of 17 volunteers collected 35 profiles, which covered a range of activities to highlight the variability of individual exposures between November 2012 and May 2013 in Scotland. They measured particulate matter by The Dylos, and combined these data with those from a GPS track stick at a private residential building with a PM_2.5_ concentration of 10.20 µg/m^3^, which was higher than in other places [27]. Broich (2010) et al. conducted their research over four weeks from 19 March 2010, to 21 April 2010, in Münster, Germany. Sixteen participants carried a measurement backpack for 24 h. Smoking and cooking emissions were the main indoor sources of PM_2.5_. For vehicles, the highest recorded concentration of PM_2.5_ was 21.70 µg/m^3^, which was detected on the bus [28].

The purpose of our study was to identify the dependence between individual exposure and dose of PM_2.5_. For this reason, we focused on determining the level of PM_2.5_ in every microenvironment (ME) and figuring out the relationship between the individual’s exposure and the dose of PM_2.5_.

## 2. Materials and Methods

### 2.1. Study Area

**Figure 1:**
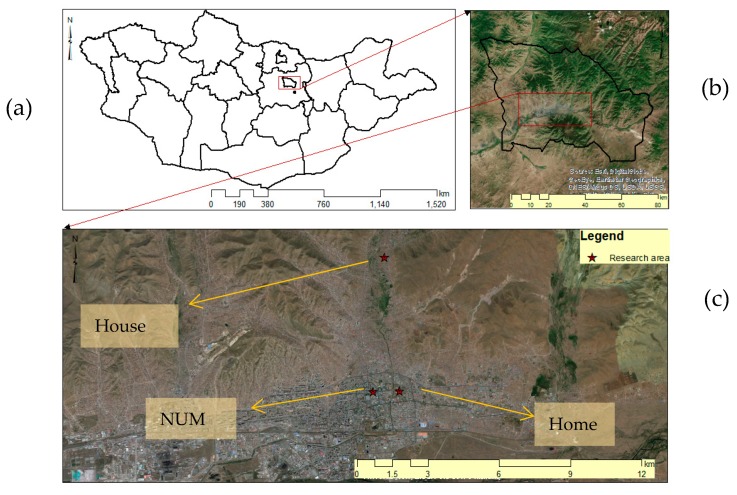
Study areas: (**a**) Mongolia; (**b**) Ulaanbaatar; and (**c**) the main microenvironments.

One of the study areas was the building of the National University of Mongolia (NUM), which is located in the center of the city. Another study area was a participant’s apartment (home 1) located 1 km away from the NUM. However, in mid-October, the participant moved to a campsite (home 2), 13 km away from the NUM (Figure 1). 

### 2.2. Study Object

A researcher (a full-time student) from the NUM cooperated as a participant in this study. According to the study, students who are enrolled at a university or a college spend 3.50 h per day in class and partaking in education-related activities [29]. The study object spent approximately 7.90 h at the NUM every single day from October to December 2018.

### 2.3. Portable Monitoring Solution

**Figure 2:**
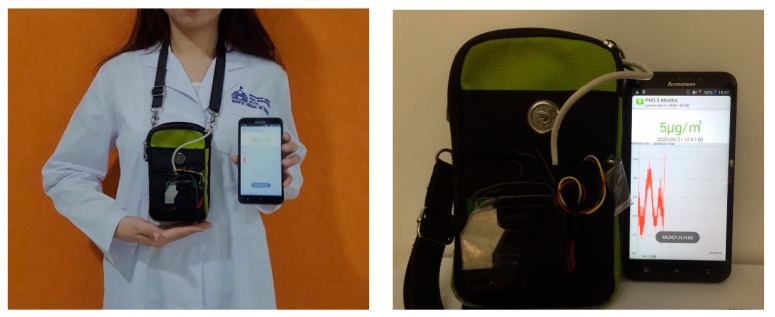
Appearance of the instrument.

Figure 2 displays the instrument designed by Nagoya University and Panasonic Corporation, Japan. The monitoring pack—the PM_2.5_ sensor—was strapped onto the study participant’s shoulder in a bag around her breathing height. The size was 52 × 45 × 22 mm, the PM_2.5_ concentration was determined by the distribution of the light-scattering technology, and the fine particle content was directly expressed in μg/m^3^. The validation of the PM_2.5_ sensor was carried out with beta attenuation monitoring (BAM) instruments (Thermo Fisher, SHARP 5030, DKK-TOA, model FPM-377, and Kimoto, model PM-712) at four urban and suburban sites in Fukuoka, Kadoma, Kasugai, and Tokyo, and the correlation factors were 0.87, 0.86, 0.86, and 0.89, respectively. For calibration, the PM_2.5_ mass concentration was calculated using the particle size of monodisperse polystyrene latex (PSL) and the particle number density measured with the condensation particle counter (CPC, TSI, model 3772). As an example of the results, the concentrations of PSL particles with diameters of 0.296 and 0.498 mm measured by the CPC were approximately 17 and 13 particles/cm^3^, respectively. The linearity of the sensor was tested using cigarette smoke particles. A test room (31 m^3^) with ten PM_2.5_ sensors and a digital dust monitor (Shibata, model LD-3B) was filled with cigarette smoke because there is no clear difference between the density of PSL particles (1.05 g/cm^3^) and the typical densities of cigarette smoke particles (1.0–1.3 g/cm^3^) [30]. 

On a full power bank, the PM_2.5_ runs for approximately 2–3 days. Furthermore, the built-in memory is able to store the data for around one year when continuously sampling logs every ten seconds per minute. The data for 8–10 days for 24 h per day were collected for each month.

### 2.4. Data Collection, Extraction, and Processing

**Figure 3:**
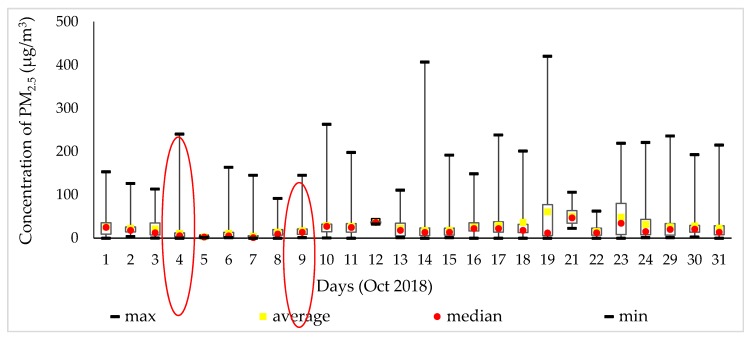
PM_2.5_ concentration with the time interval.

The sensor collected data from 1 October 2018, to 31 December 2018, and measured close to breathing height (Figure 3). The data were recorded at intervals of 10–30 s. We calculated descriptive indications, such as the minimum value, mean, median, and maximum value of the collected data. We chose days from every measured months that can represent the daily average exposure. One of the chosen days consisted of the ordinary route, and the other day consisted of a number of the microenvironments. Additionally, some statistical analyses, such as standard deviation, variance, coefficient variance, average, and median values, analyzed the result of every microenvironment (home/house, NUM, means of transportation, restaurant, pub, bar, and sports hall).

### 2.5. Data Analysis

We determined the PM_2.5_ concentration with the participant’s breath. The day–night exposure and total exposure of the participant’s daily route were calculated. There were two reasons to divide exposures into day and night.

The inhalation rate when sleeping is six times less than that in normal breathing. Therefore, the amount of PM_2.5_ in the human body decreases, and thus the study participant was assumed to be asleep during the night time [31].

The Mongolian National Standard (MNS 4585:2016) for air quality considers daytime to be from 7:00 to 22:00 and night-time is from 22:00 to 7:00 [32].

According to the study, men and women between the ages of 16 and up to the age of 21 breathe 16.3 m^3^ air per day [31]. Generally, the human inhalation rate is 16.3 m^3^/day. Consequently, the daytime inhalation rate is 0.873 m^3^/h and the night-time inhalation rate is 0.295 m^3^/h.

For the inhalation process, the exposure was estimated for each of the microenvironments in which the participant spent time and each macroactivity that would result in a different inhalation rate while engaging in that activity (Equation (1)). The exposure for 24 h was the sum of the microenvironment/macroactivity (me/ma) exposure. For each me/ma exposure, the inhalation exposure for 24 h (E_me/ma_) was defined [33,34,35].

E_me/ma_ = T_me/ma_ × C_ame_ × IR_ma_, (1)

where T_me/ma_ is the time spent in each microenvironment/macro activity (h/day), C_ame_ is the air concentration in a microenvironment (µg/m^3^), and IRma is the inhalation rate during each macroactivity (m^3^/h).

## 3. Results

### 3.1. PM_2.5_ Concentration in the Wintertime

Figure 3 shows the amount of PM_2.5_ determined by the descriptive parameters, such as the maximum, average, median, and mean values in October 2018. On 19 October, the PM_2.5_ concentration reached 420 μg/m^3^, which was the highest number that month. The daily routes of the month are represented on 4 October 2018, while 9 October represents different routes and various means of transportation compared with other days.

**Figure 4:**
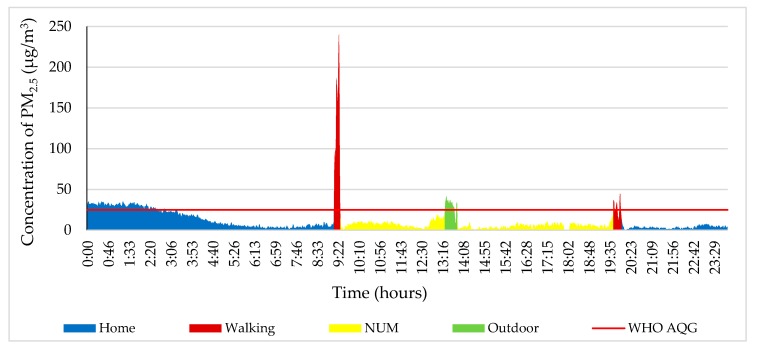
Personal profile of the participant on 4 October 2018. Each color indicates different ME and other activity.

The PM_2.5_ concentration decreased between 0:00 and 9:10 on 4 October 2018 (Figure 4). The study participant went to the NUM from home between 9:10 and 9:30. The highest concentration of the day was 230 μg/m^3^, which is 9.2 times higher than stated in the WHO air quality guidelines (25 μg/m^3^ 24-h mean) [36] due to the fact that the study object walked near the road where a street-sweeper swept up particles of dust. The level of PM_2.5_ fluctuated from 20 to 40 µg/m^3^ at the NUM from 13:10 to 13:50 while the study participant was outdoors around the NUM. However, from the NUM to home, the PM_2.5_ concentration was in a range of 10 to 36 µg/m^3^ from 19:30 to 19:50.

**Figure 5:**
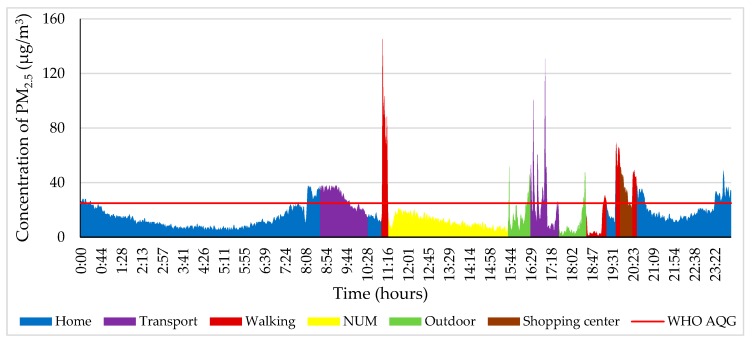
Personal profile of the participant on 9 October 2018. Each color indicates a different microenvironment (ME) and personal activity.

In Figure 5, from 0:00 to 8:40, the PM_2.5_ concentration was 7–37 µg/m^3^ at home on 9 October 2018. The study participant travelled to Modnii-2 (5 km away from the NUM to the west) by bus from 8:40 to 9:20 and then back home. At that time, the PM_2.5_ concentration increased by 20–40 µg/m^3^. The maximum amount during the day was 145 µg/m^3^, which occurred from 11:00 to 11:15, when the participant went from home to the NUM library; this is 5.80 times higher than that suggested by the WHO air quality guidelines. Thereafter, the PM_2.5_ concentration decreased slowly. From 15.40 to 16.20, the green color indicates an outdoor locality where the Music and Dance College of Mongolia is located (600 m far away from NUM). After that, the participant went to Modnii-2 (an apartment complex, 5 km away from NUM) and back home by bus. Some windows were opened on the bus; therefore, the PM_2.5_ concentration ranged from 11 to 130 µg/m^3^. From 19:50 to 20:20 around the shopping center or E-Mart (1 km away from the NUM to the east), the concentration of PM_2.5_ reached 69 μg/m^3^, which is 1.40 times higher than that suggested by the WHO air quality guidelines. 

**Figure 6:**
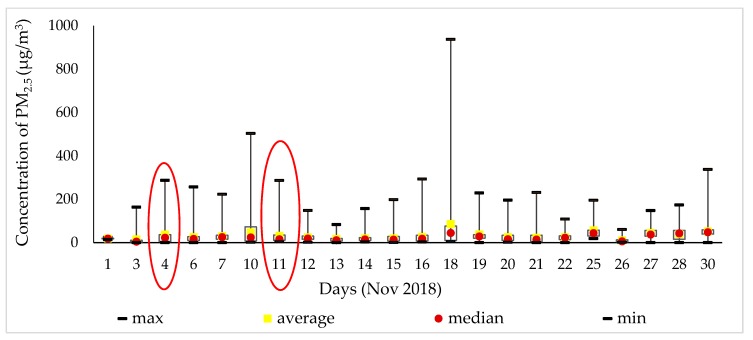
Result of PM_2.5_ concentration with the time interval.

Figure 6 illustrates that all measurements of PM_2.5_ were defined by the maximum, average, median, and mean values in November 2018. On 18 November, the PM_2.5_ concentration reached 936 μg/m^3^, which is 37.40 times higher than that suggested by the WHO air quality guidelines. Moreover, that was the highest measurement of the month. The day of 4 November 2018 chosen due to the sports center, while 11 November 2018 represents the daily route of a study participant.

**Figure 7:**
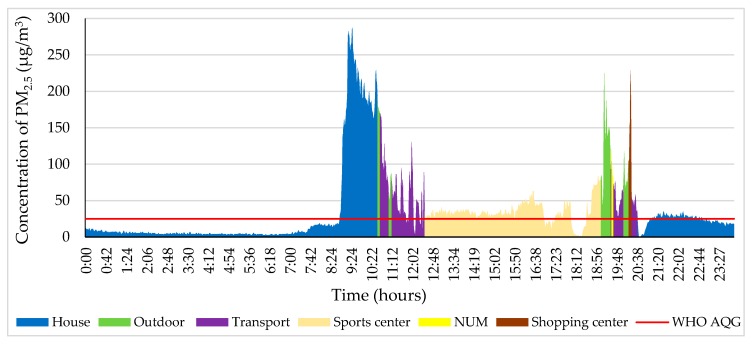
Personal profile of the participant on 4 November 2018. Each color indicates a different ME and personal activity.

As shown in Figure 7, the PM_2.5_ concentration was relatively stable until 7:40 on 4 November 2018. After that, the PM_2.5_ concentration sharply increased because the study participant wiped off a table near the measuring instrument in the house. The maximum level of PM_2.5_ was 287 µg/m^3^, which is 11.40 times higher than that suggested by the WHO air quality guidelines. The participant travelled from the house to Hunsnii-4 (750 m away from NUM to the north) from 10:35 to 11:20 by bus and then travelled to the NUM by car until 12:30. The PM_2.5_ concentration fluctuated from 13 to 77 µg/m^3^ from 12:40 to 19:00 at the sports center of the school (a bus stop, 1.3. km away from NUM to the north). Interestingly, the PM_2.5_ concentration reached 225 µg/m^3^ while the participant walked to the NUM from the sports center for 15 min. After that, the participant waited for the bus between 20:10 and 20:20 at the shopping center, where the PM_2.5_ concentration measured over 72–100 µg/m^3^.

**Figure 8:**
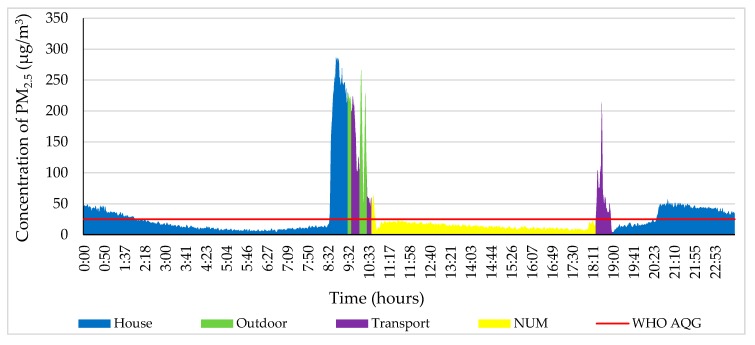
Personal profile of the participant on 11 November 2018. Each color indicates a different ME and personal activity.

The blue color represents the PM_2.5_ concentration in the house, as shown in Figure 8. At 8:35, the participant sprayed an air freshener near the measuring instrument. Therefore, the PM_2.5_ concentration strongly increased and reached 283 µg/m^3^, which is 11.40 times higher than that suggested by the WHO air quality guidelines. For that reason, this was the highest value of the day. The participant traveled to the NUM from the house from 9:30 to 10:40 by bus. She stayed at the NUM until 18:20, where the PM_2.5_ concentration was below the air quality standard. From 18:30 to 19:00, the participant traveled back to the house from the NUM by public transport. From 20:30 on this day, the concentration of PM_2.5_ was 50 µg/m^3^ at the house.

**Figure 9:**
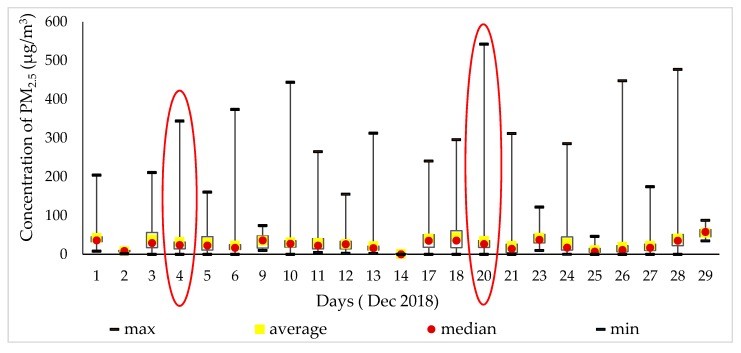
PM_2.5_ concentration in different time intervals.

As shown in Figure 9, in December 2018, the amount of PM_2.5_ was determined by the maximum, average, median, and mean values of descriptive parameters. On 20 December, 2018, the PM_2.5_ concentration reached 542 μg/m^3^, which is 20.20 times higher than that suggested by the WHO air quality guidelines. Furthermore, it was the highest level of the month. We display measurements from 4 December 2018.

**Figure 10:**
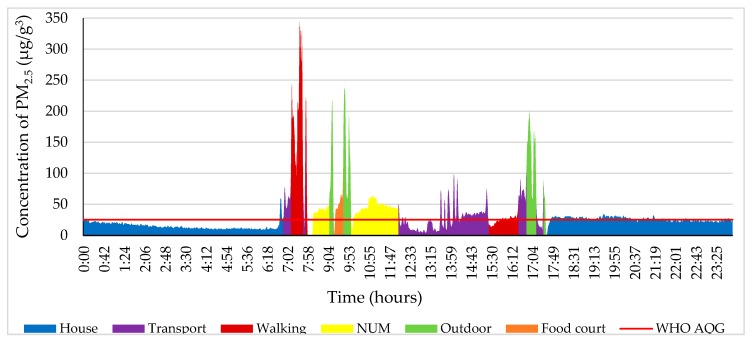
Personal profile of the participant on 4 December 2018. Each color indicates a different ME and personal activity.

Figure 10 illustrates all measurements of PM_2.5_ on 4 December 2018. The blue color shows the PM_2.5_ concentration in the house from 0:00 to 7:00. From 7:10 to 7:35, the PM_2.5_ concentration was 343 µg/m^3^, which means it was 13.60 times higher than that suggested by the WHO air quality guidelines. Meanwhile, these numbers were identified as being the highest level of the day. Generally, the PM_2.5_ concentration in the NUM was higher than on the other chosen days. At the food court (400 m away from the NUM to the northeast), the level of PM_2.5_ ranged from 49 to 70 µg/m^3^. From 12:00 to 15:20, the study participant traveled to Tasganii Ovoo (the ger district, 2 km away from the NUM to the northeast), Naiman Sharga (1.40 km away from the NUM to the east), Tasganii Ovoo, and back to the NUM by car. The participant walked around the NUM while the concentration of PM_2.5_ was above the WHO air quality guidelines. After that, the concentration of PM_2.5_ fluctuated between 28 and 90 µg/m^3^ when the study participant returned home by bus. 

**Figure 11:**
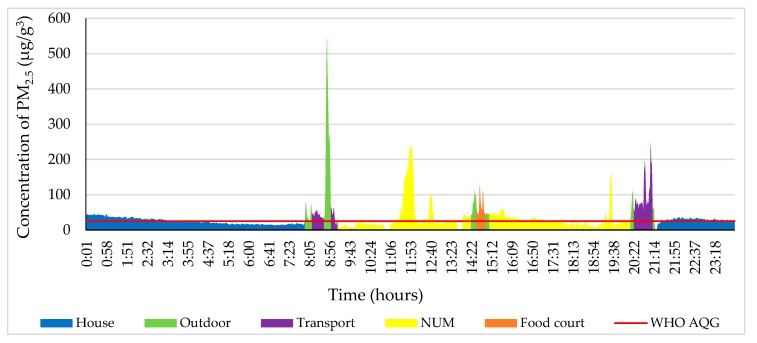
Personal profile of the participant on 20 December 2018. Each color indicates a different ME and personal activity.

From 0:00 to 7:50, on 20 December 2018, the PM_2.5_ concentration at the house is indicated by the blue color (shown in Figure 11). Between 8:50 and 9:00, while the participant was waiting for the bus at the bus stop, the measurement of PM_2.5_ reached 542 µg/m^3^, which is 20.20 times higher than that recommended by the WHO air quality guidelines, and this was the highest result of the day. The participant was in the lecture room from 9:20 to 14:20. From 14:40 to 15:00, the participant visited the food court near the NUM where the PM_2.5_ concentration reached 100 µg/m^3^, which is 2 times higher than the air quality standard. 

As shown in Table 1, the PM_2.5_ level on public transportion was higher than in other microenvironments, while the PM_2.5_ level at the karaoke bar was the lowest.

The average dose experienced by the participant for one hour or less on the selected days from every microenvironment (ME) is shown in Table 2. This table shows how high the PM_2.5_ dose that the participant received from each ME at the same time per hour. In this regard, the study participant received the maximum dose of PM_2.5_ on her body from outdoor locations and transportation.

### 3.2. Personal Exposure and Dose

On the chosen days, between 4:00 and 7:00, the exposure level of PM_2.5_ was lower than that suggested by the WHO air quality guidelines. However, personal exposure showed the highest value from 7:00 to 12:00 (Table 3). Lim et al. concluded (2018) that the UB daily profile and the PM_2.5_ concentration showed lower values at night-time, while there were increased values in the early morning, and values peaked in noon [37]. 

## 4. Discussion

### 4.1. PM_2.5_ Concentration in Various Microenvironments

The personal exposure from each microenvironment depended on the participant’s length of exposure, location, and other activities. The PM_2.5_ concentration in the home/house increased during cleaning and cooking activities [38,39,40,41]. The house was a new building and also displayed higher PM_2.5_ measurements. The average concentration of PM_2.5_ at the NUM was 26.04 ± 33.98 μg/m^3^. Furthermore, the fine particles in outdoor and indoor locations of the NUM were classified as “very strong positive” (*r* = 0.83) [42]. 

Restaurants and public transportation had the highest PM_2.5_ concentration values. We assumed that the restaurant included a fast-food restaurant, food court, and a non-smoking bar, where the frying and roasting of foods was the reason for an intensified PM_2.5_ concentration. There are 1135 public means of transportion in UB, of which 135 are 12-year-old buses and 527 are 11-year-old buses [12]. 

**Figure 12:**
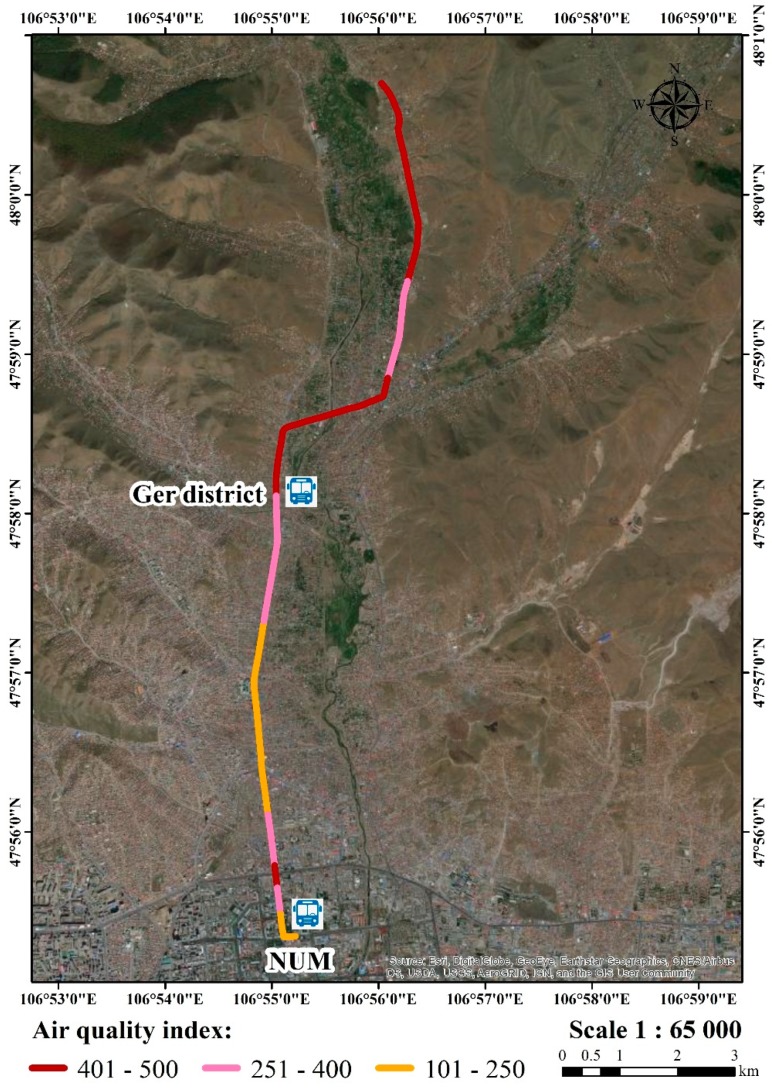
Air quality index for transportation.

Figure 12 illustrates the study participant’s route to the house from the NUM on 11 November, 2018. The air quality index is shown to aid in the understanding of what the local air quality means to human health. To make it easier to understand, the air quality index is divided into six levels of health concern in Mongolia (Table 4). 

### 4.2. Time–Activity Pattern of the Participant

For the study, the participant spent 89.01% of her time indoors and four major microenvironments were classified: (1) home/house, (2) the NUM, (3) the restaurant, and (4) other indoor locations. The most time was spent at the home/house, which represented 54.09% (631 h 7 min) of the entire study period. According to the study results, the participant spent 10.18 h per day at home. The NUM was the next major indoor location, which made up 33.33% of the day. Lastly, 1.95% of the time was spent in the restaurant and other indoor places from 1 October 2018 to 31 December 2018. However, the study participant spent 5.90% of her time on modes of transportation. The PM_2.5_ concentration of the restaurant was higher than that of other microenvironments, but the time spent there was shorter than for other indoor locations. On the other hand, the PM_2.5_ transportation level was lower than that of restaurants, while the time spent there was longer than around 1.15 h per day. Therefore, public transit will become an important issue.

### 4.3. Study Limitations

This study involved one participant and we determined her individual exposure, microenvironment, and time–activity pattern by a measuring instrument. Although this study is based on one full-time student, the collected data are being considered sufficient for analysis, comparison, and calculation. Additionally, the data collection was interrupted in some cases when the measuring tool was temporarily used in another study or when the power bank was being charged.

## 5. Conclusions

Within this study, we determined the PM_2.5_ concentration in different microenvironments and determined individual exposure values. The study included the following:

1. The data were collected from 1 October 2018, to 31 December 2018, and the participant spent most of her time indoors. In the microenvironments, the average PM_2.5_ levels were 19.50 ± 32.26 µg/m^3^ at home, 30.64 ± 36.55 µg/m^3^ at the house, 26.24 ± 33.98 µg/m^3^ at the NUM, 42.87 ± 36.17 µg/m^3^ in public transportion, and 55.73 ± 84.79 µg/m^3^ at the restaurant, respectively;
2. We estimated the exposure of PM_2.5_ for selected days. The maximum level of exposure occurred on 4 November 2018. According to the measurements of the day, the participant inhaled the maximum amount of PM_2.5_ from 09:00 to 11:00;
3. The PM_2.5_ concentration increased because of traffic congestion and burning coal at the time of starting and finishing work. In addition, the fact that road and the street are swept at that time is another reason behind the increasing concentration of PM_2.5_. In order to diminish individual exposure and reduce the conjunction of events, the street or road should be swept at another time.

## Figures and Tables

**Table 1:** Level of PM_2.5_ in various microenvironments.

Microenvironments	*N*	Maximum (µg/m^3^)	Minimum (µg/m^3^)	Mean (µg/m^3^)	SD (µg/m^3^)
Home	17,031	407.10	0	19.50	32.26
House	42,821	503.58	0	30.64	36.55
National University of Mongolia	38,662	909.16	0	26.24	33.98
Public Transportation	3988	373.80	0.14	42.87	36.17
Car	2384	98.42	0	20.46	16.54
Other Indoor	1102	263.20	0	25.16	28.59
Restaurant/Food Court/	1124	693.28	0	55.73	84.79
Sport Center	647	89.18	1.40	34.15	14.42
Karaoke	117	26.18	5.60	17.37	5.05
Pub and Bar	146	64.96	9.10	40.34	15.28

**Table 2:** Doses in various microenvironments.

Day	Doses in Various MEs (µg/m^3^)							
	Home	NUM	Means of Transportation	Outdoor	Shopping Center	Sport Center	Food Court	Sum of the Day
4 October 2018	4.20	5.20	*	17.30	*	*	*	158
9 October 2018	8.80	13.10	25.90	8.30	16.30	*	*	314.20
4 November 2018	24	*	93.70	102.20	13	29.20	*	769
11 November 2018	21	12.10	85.70	73.50	*	*	*	584
4 December 2018	11.10	39.10	27.70	76.60	*	*	34.80	637
20 December 2018	9.20	28.80	55.50	67.20	*	*	32.50	596.80

* no measurements in these microenvironments.

**Table 3:** Individual exposure and dose on selected days.

	4 October 2018		9 October 2018		4 November 2018		11 November 2018		4 December 2018		20 December 2018	
Time	Concentration of PM_2.5_ (µg/m^3^)	Dose (µg/m^3^)	Concentration of PM_2.5_ (µg/m^3^)	Dose (µg/m^3^)	Concentration of PM_2.5_ (µg/m^3^)	Dose (µg/m^3^)	Concentration of PM_2.5_ (µg/m^3^)	Dose (µg/m^3^)	Concentration of PM_2.5_ (µg/m^3^)	Dose (µg/m^3^)	Concentration of PM_2.5_ (µg/m^3^)	Dose (µg/m^3^)
0:00–0:59	32	9	23	7	9	3	44	13	21	6	42	13
1:00–1:59	31	9	15	4	6	2	32	10	18	5	36	11
2:00–2:59	26	8	11	3	5	1	21	6	14	4	32	9
3:00–3:59	20	6	8	2	5	1	14	4	12	4	26	8
4:00–4:59	12	3	7	2	4	1	10	3	11	3	22	6
5:00–5:59	6	2	7	2	4	1	7	2	11	3	17	5
6:00–6:59	4	1	10	3	4	1	7	2	19	6	15	4
7:00–7:59	3	3	19	16	9	8	10	9	142	124	18	16
8:00–8:59	6	5	31	27	42	37	76	66	30	26	104	91
9:00–9:59	39	34	31	27	237	207	216	189	88	77	19	17
10:00–10:59	9	8	17	15	166	145	81	71	39	34	15	13
11:00–11:59	7	6	31	27	57	50	20	18	50	44	85	74
12:00–12:59	5	4	16	14	36	32	18	16	17	15	39	34
13:00–13:59	22	20	11	10	34	30	15	13	21	18	22	19
14:00–14:59	3	3	9	8	32	28	13	11	36	31	61	53
15:00–15:59	3	3	9	8	34	29	11	10	28	24	45	39
16:00–16:59	6	5	25	22	47	41	10	9	63	55	31	27
17:00–17:59	6	5	15	13	29	25	8	7	45	39	24	21
18:00–18:59	6	5	9	7	27	23	47	41	29	25	14	13
19:00–19:59	12	10	26	23	91	79	14	12	28	25	30	26
20:00–20:59	4	4	33	28	52	45	32	28	27	23	68	60
21:00–21:59	2	2	15	13	28	24	47	41	25	22	44	38
22:00–22:59	3	1	16	5	27	8	43	13	23	7	32	9
23:00–23:59	5	2	28	8	20	6	37	11	23	7	28	8

**Table 4:** Air quality index values.

Numerical Values	Levels of Health Concern	Colors
When the AQI Is in This Range:	Air Quality Conditions Are:	As Symbolized by This Color:
0–50	Good	Green
51–100	Moderate	Yellow
101–250	Unhealthy for sensitive groups	Orange
251–400	Unhealthy	Pink
401–500	Very unhealthy	Maroon
+501	Hazardous	Red
